# Correlative Scanning-Transmission Electron Microscopy Reveals that a Chimeric Flavivirus Is Released as Individual Particles in Secretory Vesicles

**DOI:** 10.1371/journal.pone.0093573

**Published:** 2014-03-28

**Authors:** Julien Burlaud-Gaillard, Caroline Sellin, Sonia Georgeault, Rustem Uzbekov, Claude Lebos, Jean-Marc Guillaume, Philippe Roingeard

**Affiliations:** 1 Plate-Forme RIO des Microscopies, PPF ASB, Université François Rabelais and CHRU de Tours, Tours, France; 2 Département Bioprocess, Upstream Platform, Sanofi Pasteur, Marcy l'Etoile, France; 3 INSERM U966, Université François Rabelais and CHRU de Tours, Tours, France; UMR Inserm U1052/CNRS 5286, France

## Abstract

The intracellular morphogenesis of flaviviruses has been well described, but flavivirus release from the host cell remains poorly documented. We took advantage of the optimized production of an attenuated chimeric yellow fever/dengue virus for vaccine purposes to study this phenomenon by microscopic approaches. Scanning electron microscopy (SEM) showed the release of numerous viral particles at the cell surface through a short-lived process. For transmission electron microscopy (TEM) studies of the intracellular ultrastructure of the small number of cells releasing viral particles at a given time, we developed a new correlative microscopy method: CSEMTEM (for correlative scanning electron microscopy - transmission electron microscopy). CSEMTEM analysis suggested that chimeric flavivirus particles were released as individual particles, in small exocytosis vesicles, via a regulated secretory pathway. Our morphological findings provide new insight into interactions between flaviviruses and cells and demonstrate that CSEMTEM is a useful new method, complementary to SEM observations of biological events by intracellular TEM investigations.

## Introduction

The members of the *Flaviviridae* family are small, enveloped viruses, and include the genera *Flavivirus*, *Pestivirus* and *Hepacivirus* [1]. The genus *Pestivirus* includes the bovine viral diarrhea virus (BVDV) and the classical swine fever virus (CSFV), two animal pathogens responsible for economic losses in the livestock industry. Hepatitis C virus (HCV) is the best studied member of the genus *Hepacivirus*, as HCV infection is a major cause of chronic hepatitis, liver cirrhosis and hepatocellular carcinoma in humans, affecting 170 million people worldwide [2]. Finally, the genus *Flavivirus* comprises more than 70 viruses, many of which are arthropod-borne human pathogens causing a range of important diseases, including fevers, encephalitis and hemorrhagic fever. Flaviviruses include dengue virus (DENV), yellow fever virus (YFV), West Nile virus (WNV), Japanese encephalitis virus (JEV) and tick-borne encephalitis virus (TBEV) [1]. DENV merits particular attention, because recent investigations have indicated that this virus causes an estimated 390 million new infections worldwide each year, 96 million of which are associated with subclinical or more severe clinical symptoms, from mild fever to potentially fatal dengue shock syndrome [3].

The *Flaviviridae* genome is a single-stranded RNA molecule, which, upon its introduction into the host cell, is recognized as a messenger RNA and translated by the host cell machinery, to yield a polyprotein [1]. Processing by viral and cellular enzymes releases the individual viral gene products. The structural proteins constituting the virion consist of a core and envelope proteins. Most of the nonstructural proteins associate to form the replicase complex, which catalyzes RNA accumulation, in close association with modified host-cell membranes [4]–[9]. Several reports have described the intracellular morphogenesis of DENV, YFV and BVDV, revealing that these viruses assemble by budding at the endoplasmic reticulum (ER) membrane, leading to the accumulation of viral particles in dilated ER cisternae [4], [7], [10]–[13]. For HCV, it has proved extremely difficult to visualize the virus in infected cells [9], but an HCV-like particle model based on the production of the viral structural proteins has demonstrated that HCV also buds at the ER membrane [15]. However, the mechanism leading to the release of flavivirus virions from the infected cells remains poorly documented. It is believed that virions transit from the ER lumen to the cell surface via the secretory pathway [1], but this process is probably very rapid and has yet to be documented by microscopic approaches. In this study, we took advantage of the development of an optimized system of chimeric YFV/DENV production for vaccine purposes to study this phenomenon. We also used correlative microscopy, a powerful method for targeting and studying rare structures or rapid biological events [16]–[19]. Rather than using the well described correlative light-electron microscopy (CLEM) technique, we established a new method for this study: correlative scanning electron microscopy-transmission electron microscopy (CSEMTEM). This new type of correlative microscopy, based on the detection of cells of interest by scanning electron microscopy (SEM), for further investigation by transmission electron microscopy (TEM), made it possible to visualize the release of a flavivirus at the cell surface. Our morphological data suggest that individual viral particles are secreted from infected cells in small secretory vesicles and that this new correlative microscopy method would be useful for deciphering other biological processes.

## Materials and Methods

### Cell culture and virus infection

Vero cells (African green monkey cell line) from the Sanofi Pasteur cell bank were amplified in multitrays in a completely animal-derived component-free process (serum-free media, recombinant trypsin and soybean inhibitor). For the production step, cells were used to seed a 12-liter bioreactor containing serum-free medium and 2.5 g/l Cytodex I microcarriers (GE). Cells were amplified by incubation at 37°C, in the presence of 25% PO_2_, at pH 7.2, with shaking at 30 rpm, for three days. The cell amplification medium was then removed and replaced with prewarmed viral medium. Cells were then infected with a chimeric YFV/DENV based on the YFV 17D live attenuated vaccine backbone containing the prM and envelope genes from DENV [20]. The viral inoculum was introduced into the bioreactor at a MOI of 0.001, two hours after the medium was changed. Two days after infection, the medium was replaced. Bioreactor sampling was undertaken on a daily basis, to monitor cells and virus production.

### Analysis of the secreted particles by negative staining electron microscopy and immunogold labeling

The clarified cell-culture supernatant was ultrafiltrated (300 kDa) and the viral particles were purified by polyethylene glycol precipitation followed by an utracentrifugation on sucrose gradient. Fractions of interest were then pooled and concentrated with an Amicon – 30 kDa (Millipore) device, before to be fixed (v/v) with paraformaldehyde 2% (Sigma, St-Louis, MO), 0.1 M phosphate buffer pH 7.2 overnight. Formvar/carbon-coated nickel grids were deposited on a drop of fixed sample during five minutes and rinsed three times with phosphate-buffered saline (PBS). After a single wash with distilled water, the negative staining was then performed with three consecutive contrasting steps using 2% uranyl acetate (Agar Scientific, Stansted, UK), before analysis under the transmission electron microscope (JEOL 1011, Tokyo, Japan).

For immunogold labeling, grids coated with the sample were washed and further incubated for 45 minutes on a drop of PBS containing 1∶10 mouse monoclonal antibody against DENV E glycoprotein (DE1, Abcam, Cambridge, UK). After six washes with PBS, grids were further incubated for 45 minutes on a drop of PBS containing 1∶30 gold-conjugated (10 nm) goat-anti-mouse IgG (Aurion, Wageningen, Netherlands). Grids were then washed with 6 drops of PBS, post-fixed in 1% glutaraldehyde, rinsed with two drops of distilled water, before to be negatively stained and observed under the microscope as described above.

### Ultrastructural analysis of the infected cells by scanning electron microscopy

Chimeric YFV/DENV-infected Vero cells grown on microcarriers and were studied before infection (day 0) and every day post-infection, from day 1 to day 4. On each day, 10 ml of microcarrier suspension was fixed by incubation for 24 h in 4% paraformaldehyde, 1% glutaraldehyde in 0.1 M phosphate buffer (pH 7.2). Samples were then washed in phosphate-buffered saline (PBS) and post-fixed by incubation with 2% osmium tetroxide for 1 h. Samples were then fully dehydrated in a graded series of ethanol solutions and dried in hexamethyldisilazane (HMDS, Sigma, St-Louis, MO). Finally, the dry sample was sprinkled onto carbon disks and coated with 40 Å platinum, with a GATAN PECS 682 apparatus (Pleasanton, CA), before observation under a Zeiss Ultra plus FEG-SEM scanning electron microscope (Oberkochen, Germany).

### Ultrastructural analysis of the infected cells by transmission electron microscopy

Microcarriers were placed in a mixture of (1∶1) propylene oxide/Epon resin (Sigma) and then left overnight in pure resin for impregnation of the samples. Microcarriers were then embedded in Epon resin (Sigma), which was allowed to polymerize for 48 hours at 60°C. Ultra-thin sections (90 nm) of these blocks were obtained with a Leica EM UC7 ultramicrotome (Wetzlar, Germany), as previously described [21]. Sections were deposited on formvar/carbon-coated nickel grids and stained with 5% uranyl acetate, 5% lead citrate. Observations were made with a JEOL 1011 transmission electron microscope.

### Correlative scanning electron microscopy-transmission electron microscopy

Using SEM, we identified cells with chimeric viruses on their surface at medium or high magnification (from ×30,000 to ×60,000). Microcarriers bearing such chimeric virus-coated cells were monitored by SEM at low magnification (from ×30 to ×300). This made it possible to determine precisely the position of the microcarriers of interest over the entire disk. We mapped the entire disk, with the ZEISS multiscan module, to obtain larger images at high resolution. Images were then compared with observations of the disks under a Zeiss Stemi 2000c stereo microscope. Regions of interest were selected by cropping the carbon disk with a surgical scalpel. These selected regions were then included in resin by performing a flat embedding, before to be analyzed by TEM as described above. The location of the beads of interest was initially checked by toluidine blue staining on sequential 500 nm semithin sections. Photographs of these semithin sections were taken with a Nikon Eclipse 80i (Tokyo, Japan) equipped with a DS-Vi1 camera. Ultrathin sections (90 nm) were then cut once the center of the microcarrier of interest had been reached, allowing to specifically investigate the cells present at the upper side of the microcarrier and previously visualized by the SEM analysis. These selected sections of the microcarriers bearing chimeric virus-coated cells were deposited on formvar/carbon-coated nickel grids and stained with 5% uranyl acetate, 5% lead citrate. They were then observed in a JEOL 1011 transmission electron microscope.

## Results

### Analysis of the secreted particles by negative staining electron microscopy and immunogold labeling

**Figure 1:**
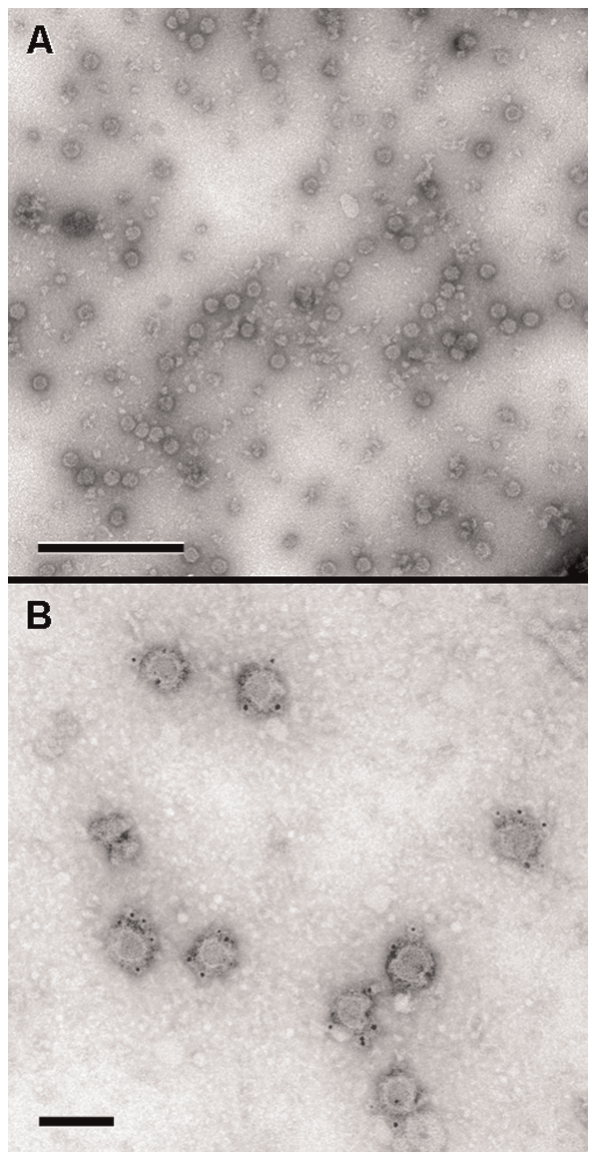
Analysis by negative staining electron microscopy and immunogold labeling of the chimeric yellow fever virus/dengue virus (YFV/DENV) particles secreted in the cell supernatant. (A): Numerous 50 to 60 nm spherical particles that had morphological characteristics of a typical flavivirus were observed by regular negative staining electron microscopy. (B): The specificity of these particles was confirmed by immunogold labeling with an anti-DENV E glycoprotein. Scale bars : 0.5 µm in A ; 100 nm in B.

Initial analysis by regular negative staining electron microscopy of the purified viral particles led to the observation of numerous spherical particles, 50 to 60 nm in diameter, that had the morphological characterisitics of a typical flavivirus [14] (Fig. 1A). The specificity of these structures was confirmed by immunogold labeling with the anti-DENV E glycoprotein antibody, showing unambiguously the presence of the DENV envelope glycoprotein at the surface of these viral particles (Fig. 1B).

### Ultrastructural analysis of the infected cells by scanning electron microscopy

**Figure 2:**
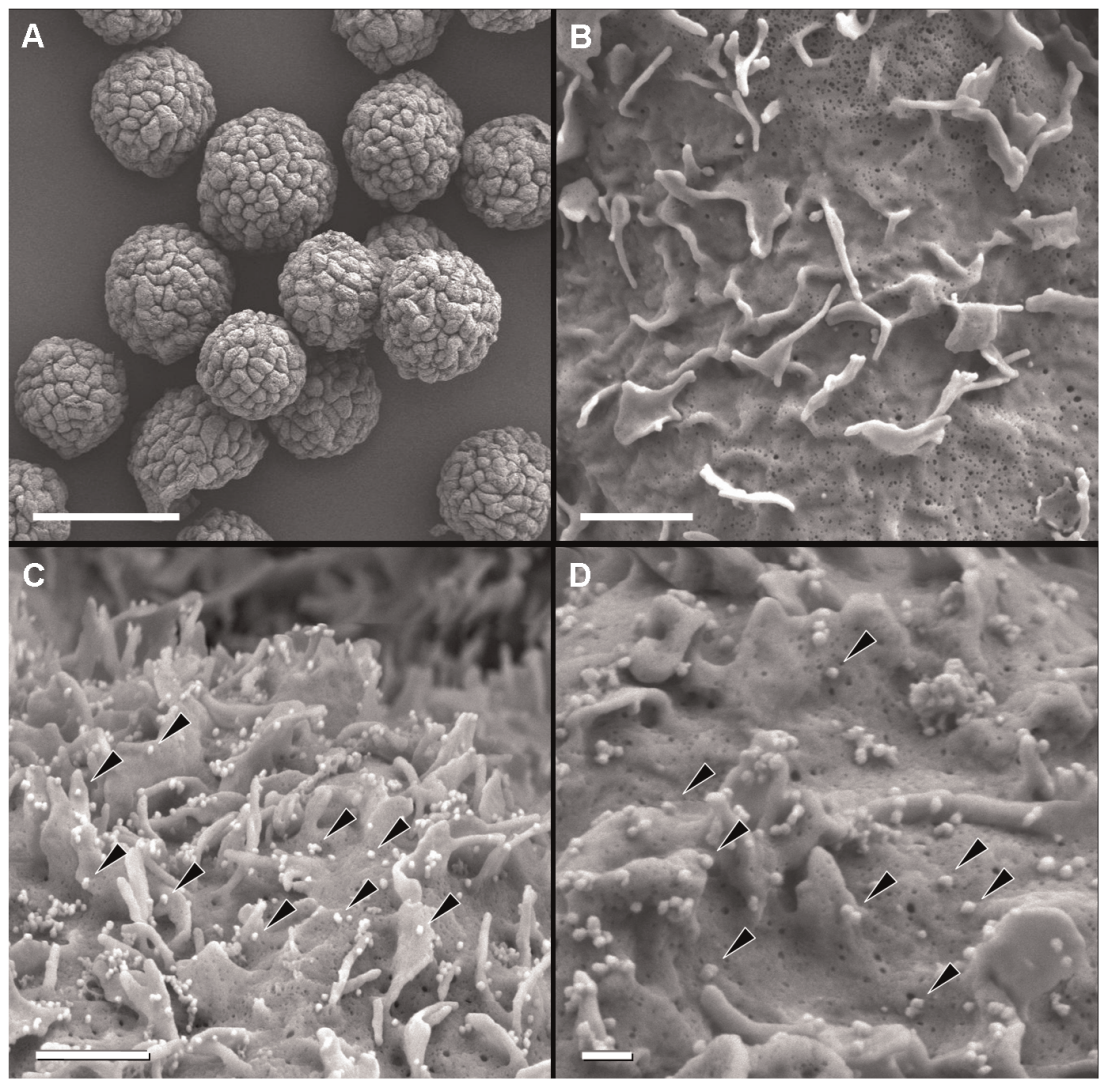
Analysis, by scanning electron microscopy (SEM), of Vero cells grown on microcarrier beads and infected with the chimeric YFV/DENV. (A): At low magnification and before infection (day 0), Vero cells completely covered the microcarrier surface. (B): On day 0, analysis of these cells at high magnification showed them to have a smooth surface with protrusions. (C and D): On day 2 post-infection, several cells presented numerous chimeric viral particles at their surface (arrows). Scale bars : 100 µm in A ; 1 µm in B and C; 0.2 µm in D.

Low-magnification SEM was used to observe the Vero cell monolayer on the microcarrier surface (Fig. 2A). These observations showed that the cells adhered efficiently to the surface of the beads, which were found to be largely covered by the cells (Fig. 2A). At high magnification and before infection, the surface of the Vero cells was smooth, with small protrusions and no detectable virus-like structures (Fig. 2B). Similar results were obtained for observations made on the day after infection with the chimeric YFV/DENV (not shown). Two and three days post-infection, some cells had large numbers of chimeric viral particles, 50 to 60 nm in diameter, at their surface (Fig. 2C and 2D). In cells displaying such particles, a large surface of the cell including protrusions was covered by the chimeric viral particles, without no evidence of any particular polarization for viral particle release or morphological modification. However, cells bearing chimeric YFV/DENV particles at their surface were very scarce. A full screening of 100 microcarriers, accounting for the observation of 2300 cells, showed that only 6 cells (0.26%), detected on 6 different microcarriers, exhibited this pattern of abundant viral release. These structures were highly specific, as none were observed in uninfected, control cells. Analyses of the Vero cells four days post-infection showed an absence of chimeric viral particles at the cell surface, despite extensive searches (not shown).

### Ultrastructural analysis of the infected cells by transmission electron microscopy

**Figure 3:**
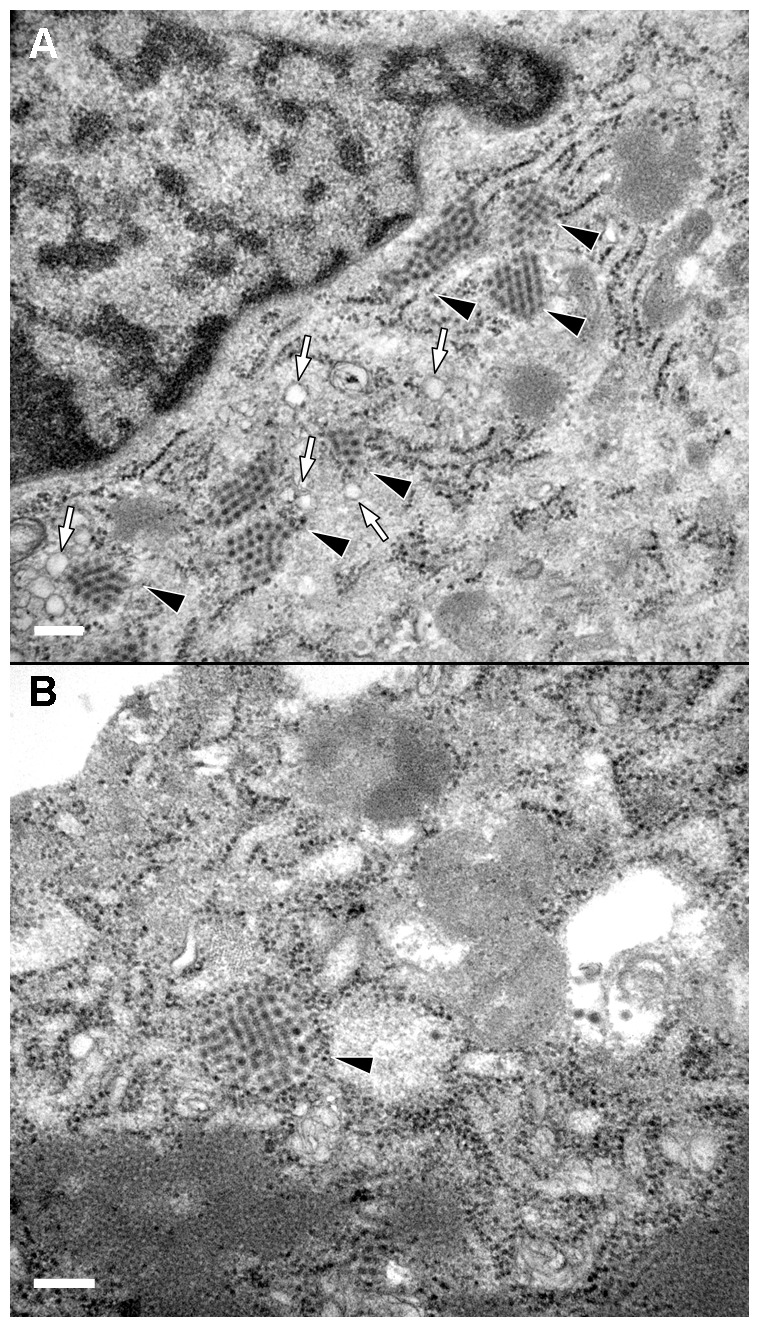
Analysis, by transmission electron microcopy (TEM), of Vero cells grown on microcarrier beads and infected with the chimeric YFV/DENV. (A and B): Ultrathin sections of the cells attached to the microcarriers showed the presence of numerous chimeric viral particles in cisterns (black arrows) linked to the rough endoplasmic reticulum. Some virus-induced smooth vesicles were observed close to rough ER cisternae containing chimeric YFV/DENV particles (white arrows). Scale bars: 0.2 µm in A and B.

In analyses of ultrathin sections of Vero cells before infection or one day post-infection with the chimeric YFV/DENV, no viral particles were visualized (not shown). From days 2 to day 4 post-infection, specific ultrastructural changes associated with the presence of chimeric YFV/DENV and described elsewhere for DENV [7], [13], [14] were easily observed (Figure 2). These changes included dilated ER-derived cisternae containing viral particles, often arranged in regular arrays (Fig. 3A and 3B) and small clusters of smooth virus-induced vesicles (Fig. 3A). The interior of these virus-induced vesicles was recently shown to be connected to the cytosol and to constitute the probable site of viral RNA replication [7]. Extensive observations of ultrathin sections of these cells between days 2 and day 4 post-infection did not result in the visualization of chimeric YFV/DENV particle release.

### Correlative scanning electron microscopy-transmission electron microscopy

**Figure 4:**
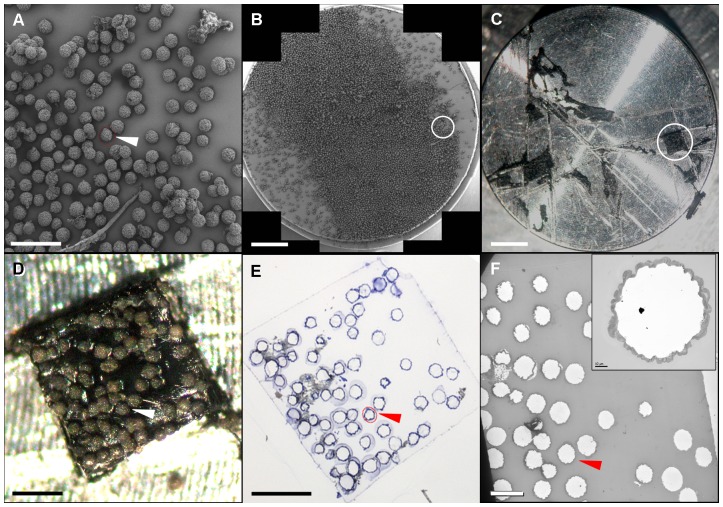
Principle underlying correlative scanning electron microscopy-transmission electron microscopy (CSEMTEM). (A): SEM acquisition, at low magnification, made it possible to locate a microcarrier of particular interest (white arrow), i.e. bearing a cell with chimeric viral particles at its surface. (B): Mapping of the whole carbon disc with the multiscan module was performed, to identify the domain of interest on the disk (white circle). (C and D): After resizing (white circle in C), the sample containing the microcarrier of interest was examined under a stereo microscope (D). (E): After inclusion in Epon resin, thin sections (500 nm) of the resized sample were cut with an ultramicrotome and stained with toluidine blue, making it possible to identify the microcarrier of interest by light microscopy. (F): Ultrathin sections (70 nm) of this block were then cut for analysis of the resized sample by TEM, making it possible to visualize cells at the surface of the selected microcarrier (inset in F). The microcarrier of interest (arrows in A, D, E and F) can be followed during all these steps, through the recording of SEM, optical and TEM photographs. Scale bars : 200 µm in A, D and E; 2 mm in B and C; 100 µm in F.

**Figure 5:**
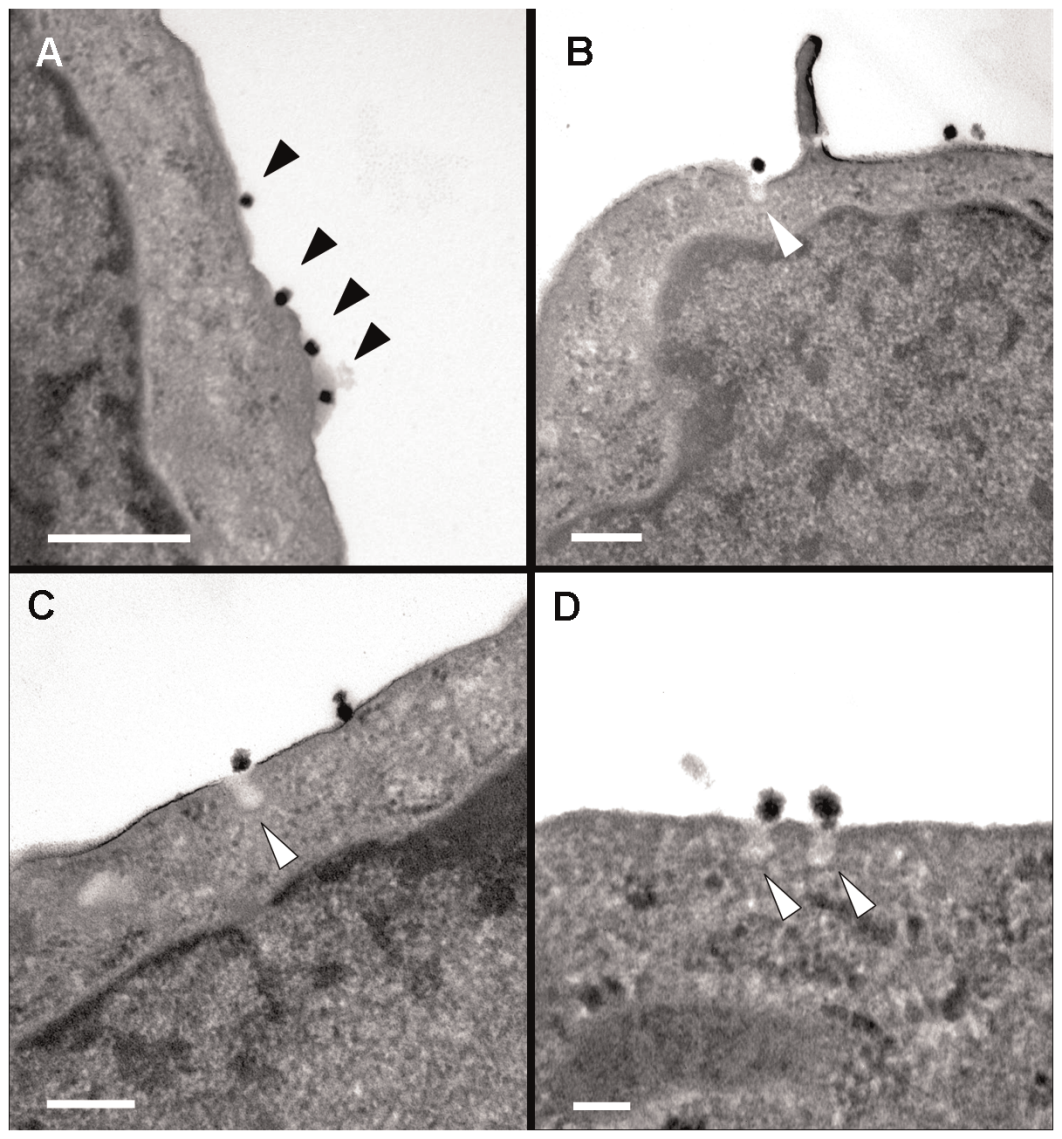
TEM analysis of Vero cells selected by the CSEMTEM method. (A): Electron-dense chimeric viral particles were observed at the cell surface (black arrows). (B, C and D): Several released particles were observed at the site of small-vesicle exocytosis at the plasma membrane (white arrows). Scale bars: 0.2 µm in A, B and C; 0.1 µm in D.

Samples of cells taken two days post-infection and initially analyzed by SEM were used for this CSEMTEM experiment. The observation of these samples at high magnification made it possible to localize several microcarriers bearing cells with numerous chimeric viral particles at their surface. Low magnification analyses of these beads of interest were used to map their neighborhood pattern (Fig. 4A), and the Multiscan module was then used to record and reconstitute the whole SEM sample (Fig. 4B). Selected acquisition parameters applied to this 6×9 digital microphotograph map provided sufficient detail, with rapid acquisition, and resulting in a final file of reasonable size (i.e. not too heavy to handle). This large image was then compared with the carbon disk observed directly under the stereo microscope. The global pattern of the beads facilitated recognition of the zone of interest and, finally, the microcarrier of interest. A surgical scalpel was then used to cut and remove the carbon disk visualized under the stereo microscope, resulting in the visualization of an area of less than 1 mm^2^ on the SEM sample support (Fig. 4C). Rapid control at the maximum magnification of the stereo microscope confirmed the localization of the microcarrier of interest in this selected portion of the SEM sample (Fig. 4D). This resized sample was embedded in Epon resin and sectioned with an ultramicrotome, to generate semi-thin sections that were stained with toludine blue and observed under a light microscope, to follow the bead of interest (Fig. 4E). Finally, several ultrathin sections of this resized sample were examined by TEM, to visualize the bead of interest at low (Fig. 4F) and high (insert in Fig. 4F) magnifications. Dextran-based microcarriers were not embedded in resin, resulting in these microcarriers appearing as holes (corresponding to the space left by the beads) surrounded by a monolayer of cells on semithin and ultrathin sections (Fig. 4E and 4F). For this reason, ultrathin sections were deposited on formvar membrane, to prevent the movement and tearing of the sections under the electron beam. The observation, by TEM, of these ultra-thin sections containing the selected cells, led to the visualization of numerous chimeric YFV/DENV particles at the cell surface (Fig. 5). The appearance of the chimeric viruses on the cell surface differed from that on regular TEM, due to the platinum coating step used in SEM analysis. Indeed, chimeric viral particles appeared to be 60 to 70 nm in diameter and very dense (Fig. 5). Clusters of these dense viral particles were frequently found at the cell surface, but some were clearly associated with an exocytosis-like pattern (Fig. 5B, 5C, 5D), suggesting that the chimeric viral particles were released individually by the exocytosis of a small secretory vesicle. Only few intracellular viral particles were observed in these virus-covered cells as compared to the surrounding cells, suggesting the occurrence of a massive release of the chimeric viruses in these particular cells. To quantify the presence of intracellular viral particles in the surrounding cells, we examined carefully 200 cells on ultrathin sections obtained with the CSEMTEM method and determined that 24 (12%) contained viral particles, often arranged in regular arrays.

## Discussion

The mechanism underlying flavivirus release from the host cell remains unclear. As these viruses accumulate in dilated ER-derived cisternae, it has been suggested that they may be released by the exocytosis of these large virion-containing vacuoles and/or as individual viral particles in secretory vesicles [12]. However, it has been also suggested that WNV could be released by a budding at the apical surface of the plasma membrane [22]. We report here the first visualization, by SEM, of a chimeric flavivirus at the surface of an infected cell. Our SEM photographs of chimeric YFV/DENV-infected cells demonstrate that the viral particles are not released in clusters in a polarized area of the cell. Instead, they are released individually and evenly over a large surface of the cell. The scarcity of chimeric virus-covered cells suggests that this phenomenon is short-lived, probably accounting for the difficulties encountered in attempts to observe the release of viral particles in ultrathin sections analyzed by TEM. This led us to develop an original method — CSEMTEM, for correlative scanning electron microscopy-transmission electron microscopy — for identifying virus-covered cells and selecting them precisely by SEM. These cells were then embedded in resin and sectioned, for further analysis by TEM. The ultrastructure of the cells that had previously been prepared for SEM analysis was found to be very well preserved when these cells were analyzed by TEM. No major difference could be found between these cells and those prepared by regular TEM protocols (i.e. without HMDS treatment and platinum coating). The major difference concerned the chimeric viral particles at the cell surface, which appeared as extremely dense particles with a diameter of 60 to 70 nm. This particular feature of the virions on CSEMTEM observation was due to the platinum coating used for the initial SEM sample preparation, resulting in the incorporation of large amounts of metal into these small objects. We tested several alternative strategies, to try to prevent this effect, including a carbon coating, but none gave satisfactory SEM observations of the cell surface and final visualization of the chimeric viral particles (not shown).

Nevertheless, our CSEMTEM method provided the first observation of a chimeric flavivirus being released as an individual particle in small exocytosis vesicles. These results are consistent with recent gene silencing experiments showing that host cell exocytosis factors, such as Sec3p and EXO70, are essential for DENV egression or secretion [23]. This is also consistent with the maturation of flavivirus particles in the Golgi compartment. Hepacivirus and pestivirus virions are infectious immediately, or at least very shortly after their envelopment [24], [25], but flavivirus particles remain immature until the acid-induced rearrangement of their envelope E protein and the furin-mediated cleavage of their prM protein have occurred in the late Golgi compartment [26], [27].

The scarcity of virus-coated cells at any given time suggests that this exocytosis is probably a very short-lived process. Moreover, the presence of large numbers of chimeric flavivirus particles evenly distributed over a large surface of these rare cells suggests that this mechanism is driven by many exocytosis vesicles being generated at the same time in a given cell. This suggests that the release of the chimeric virions occurs via a regulated exocytosis that may also account for the absence of morphological changes or obvious cytotoxicity in the virus-coated cells studied by SEM.

Our data obtained with microscopic approaches may provide new insight into basic flavivirus/cell interactions and may facilitate the definition of targets for the development of preventive and therapeutic strategies for combating infections due to these viruses. However, it will be necessary to confirm these observations with wild-type flavivirus strains. It will be also necessary to further investigate this phenomenon by confirming our morphological data with biological experiments. Nevertheless, our findings suggest that CSEMTEM is a potentially useful new correlative microscopy method for analyzing the intracellular ultrastructure of cells presenting particular surface modifications, which could be applied to the study of other important biological processes. Although limited by technical constraints, CSEMTEM will be particularly useful as compared to CLEM to study virus/cell interactions, as fluorescence methods do not reveal detailed information about the structure of viruses. Viruses can be visualized as small spots on fluorescence microscopy, but the resolution of this technique is too low to determine whether these fluorescent spots correspond to assembled virions or aggregated viral proteins. Thus, CSEMTEM will remain a useful technique for visualizing structured virions at the surface of infected cells and investigate the intracellular ultrastructure of virion-producing cells.
